# Genetic diversity and recent ancestry based on whole-genome sequencing of endangered Swedish cattle breeds

**DOI:** 10.1186/s12864-024-09959-9

**Published:** 2024-01-22

**Authors:** Ajith Harish, Fernando A. Lopes Pinto, Susanne Eriksson, Anna M. Johansson

**Affiliations:** Department of Animal Breeding and Genetics, Swedish University of Agricultural Sciences, 75007 Uppsala, Sweden

**Keywords:** Cattle, WGS, Population structure, mtDNA

## Abstract

Several indigenous cattle breeds in Sweden are endangered. Conservation of their genetic diversity and genomic characterization is a priority.

Whole-genome sequences (WGS) with a mean coverage of 25X, ranging from 14 to 41X were obtained for 30 individuals of the breeds Fjällko, Fjällnära, Bohuskulla, Rödkulla, Ringamåla, and Väneko. WGS-based genotyping revealed 22,548,028 variants in total, comprising 18,876,115 single nucleotide polymorphisms (SNPs) and 3,671,913 indels. Out of these, 1,154,779 SNPs and 304,467 indels were novel. Population stratification based on roughly 19 million SNPs showed two major groups of the breeds that correspond to northern and southern breeds. Overall, a higher genetic diversity was observed in the southern breeds compared to the northern breeds. While the population stratification was consistent with previous genome-wide SNP array-based analyses, the genealogy of the individuals inferred from WGS based estimates turned out to be more complex than expected from previous SNP-array based estimates. Polymorphisms and their predicted phenotypic consequences were associated with differences in the coat color phenotypes between the northern and southern breeds. Notably, these high-consequence polymorphisms were not represented in SNP arrays, which are used routinely for genotyping of cattle breeds.

This study is the first WGS-based population genetic analysis of Swedish native cattle breeds. The genetic diversity of native breeds was found to be high. High-consequence polymorphisms were linked with desirable phenotypes using whole-genome genotyping, which highlights the pressing need for intensifying WGS-based characterization of the native breeds.

**Supplementary Information:**

The online version contains supplementary material available at 10.1186/s12864-024-09959-9.

## Background

The application of whole-genome sequencing (WGS) based methods for analyzing the viability and preservation of livestock populations is becoming increasingly feasible. Conservation genomics methods are applied to assess genetic diversity, demographic history and degree of hybridization as well as range of analyses to assist genomic selection approaches [1]. Although cryopreservation is an integral part of the cattle breeding industry, it is largely limited to preservation of spermatozoa of a relatively small number of individuals from selected breeds for artificial insemination [2]. The small populations of indigenous Swedish cattle breeds highlight the need to carefully monitor the use of live breeding animals, in addition to keeping cryopreserved sperm samples, to reduce the risk of inbreeding and loss of genetic diversity.

Native Swedish cattle breeds have traditionally been kept mainly as dairy cattle, and are well adapted to the Nordic climate [3, 4]. The phenotypes, different names used for the breeds and the current population size and usage of six endangered Swedish native cattle breeds included in this study are described in Table 1. The first breed associations for the two breeds Rödkulla and Fjällko were formed more than 100 years ago, but similar type of cattle had then already been present in Sweden for a long time. These two breeds were merged in a breed called Swedish Polled cattle and got a common herd book in 1938, but the crossbreeding between the former separate breeds were limited, and they are now considered as separate breeds again with new separate breed associations. Both these breeds had a much higher population size in the past and have decreased in numbers due to replacements by Swedish Red and Swedish Holstein. The Rödkulla is a polled breed that originates from the middle part of Sweden and most of them have solid red coat color, but some are red with white markings. The Fjällnära cattle breed was in the 1980s recognized as a subgroup of Fjällko. Both Fjällko and Fjällnära are polled and originates from northern Sweden. Typical colors for Fjällko and Fjällnära are white speckled or colour-sided with red, black or grey markings, and only rarely solid red, black or grey. The native breeds Väneko, Ringamålako and Bohuskulla were all discovered and recognized as old local breeds in the south or south-west of Sweden in the 1990s. The Bohuskulla is polled and shows similar coat colors and pattern as Fjällko and Fjällnära, whereas the Ringamålako commonly is red with white markings and Väneko often is white speckled or color sided, but solid color occurs. Both Väneko and Ringamålako have horns. The current population sizes of the native Swedish breeds are relatively small (Table 1). All these local breeds were traditionally kept as dairy cows and Fjällko is still often kept as a dairy cow, whereas the other breeds are now often kept as suckler cows.

**Table 1:** Information about the studied Swedish breeds. The number of individuals in each breed is för the year 2021 as reported in the DAD-IS database (https://www.fao.org/dad-is/en/)

Breed name in this manuscript	Other names that have been used for the breed	Number of animals	Coat colour	Horn/polled	Utilization
Fjällko	Fjällras, Svensk fjällras, Fjäll, Swedish mountain cattle, Swedish highland cattle	7802	Most are white with red or black and more rarely grey pattern, often colour-sided but sometimes speckled in other patterns. Some are solid red, black or grey	polled	Dairy breed, often used for production of local cheese due to favourable milk protein composition. Some are used as suckler cows
Fjällnära	Fjall cattle, Unique Swedish mountain cattle	300	Similar as Fjällko, but with a larger proportion of non-white animals	polled	Milked for subsistence farming or used as suckler cows on natural pastures, but some are milked for subsistence farming
Bohuskulla	Bohus poll	136	Similar as Fjällko	polled	Mainly used as suckler cows on natural pastures, but some are milked for subsistence farming
Rödkulla	Swedish red polled	944	Most are solid red, but some have white markings	polled	Mainly used as suckler cows on natural pastures, but some are milked for subsistence farming
Ringamålako	Ringamala cattle	122	Red with white markings	horn	Mainly used as suckler cows on natural pastures, but some are milked for subsistence farming
Väneko	Vane cattle	280	Often red with white markings, some are colour-sided. Sometimes black colour	horn	Mainly used as suckler cows on natural pastures, but some are milked for subsistence farming

In a recent study, signatures of selection in Swedish native cattle breeds were identified in genes associated with several traits including body size, cold acclimation, resistance to bacterial and parasitic infections and polled phenotype [3]. Native breeds from northern Sweden differ genetically from the breeds in the south [4]. The cattle originating from northern Sweden are instead closely related to cattle in Norway, Finland and Iceland [5–7]. This indicates that present day northern breeds share common origin in the past and are similar to the cattle that were brought from Norway to Iceland more than 1000 years ago. Within the Swedish northern cattle breeds, there is a genetic differentiation between Fjällko and the Fjällnära cattle, as well as between different subpopulations of the Fjällnära cattle [8]. The genetic differences may be due to several reasons; a since long geographic isolation, different preferences in the selection made by humans, and adaptations to the local environment, and may partly reflect diverse origins of northern Fennoscandian breeds (possible admixture among the ancestral breeds). There are some indications that cattle may originally have arrived in Sweden both from the south and from the east. Archaeological evidence and historical sources suggest that agriculture spread to Northern Sweden from the south during the historical period [9], whereas studies of genetic variation in cattle breeds suggest that some of the northernmost Nordic native cattle breeds may have a common eastern origin [6, 10].

Efforts to conserve native breeds have received attention in the recent years, both because of the importance for genetic diversity, but also for the cultural values of such breeds. Due to the decreasing costs, WGS projects have become more accessible tools for genetic analysis to provide important information of the genetic diversity of cattle populations, and thereby help conservation and breeding efforts [11]. In addition, the detection of deleterious alleles and the high-resolution analysis of mutations that is possible using WGS-based methods will improve the design and implementation of genetic tests routinely employed for screening genetic disorders in cattle breeds.

Although SNP array-based analyses of cattle populations is cost effective, the previous analyses of these local breeds were limited to 112,000 SNPs that average to about one SNP per 20 kb of the genome. In contrast, the extremely high nucleotide-level resolution using WGS-based genotyping and reconstruction of ancestral genotypes can be more informative in resolving these ambiguities resulting from low-resolution coverage with SNP chips. The aim of this study was to analyse the genetic diversity of endangered Swedish local cattle breeds on the whole genome scale. To this end we selected 30 animals for whole-genome sequencing (WGS) based on prior knowledge about genetic diversity and population differentiation from SNP-array analyses [4, 8]. These selected samples provide a good representation of the diversity of the Swedish native cattle breeds. Since coat colour is a distinctive trait for several of these breeds, we also specifically analysed variation in some coat colour genes.

## Methods

### DNA sequencing and processing

We used frozen genomic DNA from 30 individuals representing Fjällko (7 cows), Fjällnära (3 cows and 1 bull, one each from the founder herds Funäsdalen, Klövsjö, Lillhärjåbygget, and Biellojaure), Bohuskulla (2 cows and 1bull), Rödkulla (9 cows), Ringamåla (1 cow and 1 bull, both from the founder herd Rögnaröd) and Väneko (3 cows and 2 bulls) for WGS runs. The 30 individuals included in the present study were born between 1986 and 1995, and can be broadly grouped into northern (Fjällko, Bohuskulla and Fjällnära) and southern (Rödkulla, Ringamåla and Väneko) breeds. The samples were selected so that we had a higher number of samples from the breeds with higher population sizes and a longer population history (Fjällko and Rödkulla) and fewer from the breeds that are founded from local populations found in the 1990s with small population size. We also selected animals, among our samples with enough DNA for whole genome sequencing, that were born on different farms (the very few that were born on the same farm were not closely related). Based on our previous studies of the genetic diversity and population differentiation from SNP-array analyses [4, 8], the set of 30 individuals selected here is a good representation of the diversity of the Swedish native cattle breeds. For example, from the Fjällnära where the four founder herds are genetically differentiated according to a previous study [8], we selected one sample from each founder herd.

Sequencing libraries were prepared from 1 µg DNA using the Illumina TruSeq PCRfree DNA sample preparation kit targeting an insert size of 350 bp. The library preparation was performed according to the manufacturers’ instructions. The specified kit and protocol is an accredited method. Sequencing was performed using NovaSeq S4 flowcells with v1 sequencing chemistry, resulting in paired-end 150 bp read length. Sequencing was performed by the SNP&SEQ Technology Platform in Uppsala. The facility is part of the National Genomics Infrastructure (NGI) Sweden and Science for Life Laboratory.

FASTQ read were processed for a reference-guided assembly according to the guidelines prescribed by the 1000 Bull Genomes Project consortium, Run 8 [11]. Briefly, FASTQ reads were quality filtered using Trimmomatic v3.8 [12] to exclude reads with mean qscore less than 20 and length less than 35 bp. Quality controlled reads were aligned to the bovine reference genome ARS-UCD1.2_Btau5.0.1Y using the BWA-MEM algorithm of the Burrows-Wheeler Aligner [13] to produce a reference-guided assembly of genomes for each animal. This reference has the Btau5.0.1 Y chromosome assembly from Baylor College [14] added to ARS-UCD1.2 [15]. Mapped sequences were further processed to mark PCR/optical duplicates and base quality recalibration was performed according to GATK best practices [16] guidelines to prepare the genome sequences for WGS-based genotyping.

### Variant calling

We used Genome Analysis Toolkit (GATK) v4.8.1 to identify SNP and Indel poplymorphisms. We followed the workflows and parameters recommended by GATK best practices guidelines for SNP and indel calling. First GATK HaplotypeCaller was used for downstream processing of the reference-guided assemblies. The HaplotypeCaller calls SNPs and indels simultaneously following a local de-novo assembly of the haplotypes in an active region. Genomic Variant Call Format (GVCF) files were created for each genome to identify potential polymorphisms. Individual GVCF files were merged into a multi-sample GVCF file for using the GenotypeGVCFs tool, which generated a final jointly genotyped Variant Call Format (VCF) file.

### Variant evaluation, annotation and consequence prediction

The called variants were then filtered with VariantFiltration tool. For SNPs the following filtering thresholds were used (QD < 2.0, QUAL < 30.0, SOR > 3.0, FS > 60.0, MQ < 40.0, MQRankSum < -12.5, ReadPosRankSum < -8.0); and for indels the thresholds were (QD < 2.0, QUAL < 30.0, FS > 200.0, ReadPosRankSum < -20.0). Finally, biallelic variants were separated from multiallelic variants. For all analyses, biallelic variants were used unless otherwise mentioned. Summary statistics of the identified variants such as the total number of variants, number of types of variants, etc., was estimated with VCFtools v0.1.16 [17] and snpEff v5.0 [18] from the total cohort before separating biallelic variants. Novel variants were identified using Picard v2.23.6 [19] after comparing with known variants compiled by the 1000 Bull Genomes consortium. Functional consequences of the all variants, including multiallelic variants, were predicted according to the Ensemble annotation (release 99) of the bovine genome ARS-UCD1.2 using SnpEff with default parameter settings. SnpEff [18] determines the effect-impact of the predictions as HIGH, MODERATE, LOW and MODIFER, depending on whether or not a variant effect is deleterious. To verify predicted variant-impacts we further analyzed genes responsible for coat color phenotypes that are known to have been targets of selection based on other studies [20–22].

### Genetic differentiation and relationship estimates

We estimated nucleotide diversity (π) over the complete autosomal genome based on biallelic SNPs using VCFtools measured in nonoverlapping windows of 1 Mb. π was estimated as the average number of nucleotide differences per site between two genome sequences. π was quantified separately for the breeds of northern (Fjällko, Bohuskulla and Fjällnära) and southern (Rödkulla, Ringamåla and Väneko) origin as distinct populations. Additionally, π was estimated separately for genic and non-genic regions. The difference in π values were compared using box plots and diamond plots and the difference between the means of the two populations (northern and southern) were compared using unpaired two-sample t-tests.

We used principal components (PCA) analysis implemented in Plink 2.0 [23] to determine the population structure. A genetic relationship matrix was constructed from all autosomal SNPs (~ 19 million) followed by a full eigendecomposition on the matrix to infer axes of genetic variation. SNPs were not pruned for linkage disequilibrium (LD), minor alleles or other criteria for filtering of SNPs. Instead, we used four random subsets of 100,000 SNPs to check the consistency of the inferred population structure. The first two eigenvectors/principal components (PCs) were plotted to infer the population structure.

To estimate genealogy from nuclear DNA, all autosomal biallelic SNPs were used. We first used vcf2phylip v 2.0 to convert the VCF matrix to a multiple sequence alignment (MSA) matrix of variants in nexus format, suitable for phylogenetic tools. Various genetic distances were estimated from the MSA matrix: observed genetic distance (uncorrected P distance), GTR distance, TN (Tamura-Nei) distance in PAUP v 4.0a 169 to construct a neighbor-joining (NJ) tree. We also estimated a genealogy under the coalescent model using SVDquartets in PAUP [24]. Trees were visualized in FigTree v 1.4.4.

To determine patterns of maternal inheritance, we used the complete mitochondrial DNA (mtDNA) and constructed a median joining haplotype network using PopART [25]. In addition, nucleotide diversity (π) and the number of segregating sites were calculated, also in PopArt. To get a clearer picture of the maternal ancestry, specifically to identify the oldest known maternal ancestor of an individual, we examined the pedigree for Fjällko, Fjällnära, and Rödkulla samples in the database maintained by the breed association Svensk Fjällrasavel (https://db.Fjallko.se). For the breeds Bohuskulla, Ringamåla and Väneko we did not have complete pedigree information, but we obtained information from the breed association about oldest known maternal ancestor “cow line” for the samples used in this study.

## Results

### Overview of genomic variation

**Fig. 1:**
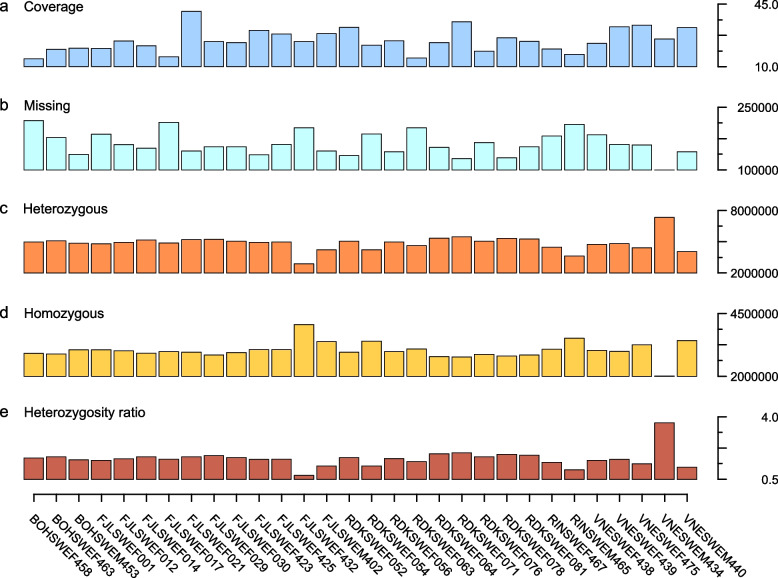
Average genome coverage and summary of detected genotypes per studied individual of 30 individuals representing Swedish native cattle breeds. Legend: **a** Average genome coverage for each individual. **b** Number of sites for which no genotype was identified. **c** Number of heterozygous genotypes. **d** Number of nonreference homozygous genotypes. **e** Heterozygosity ratio calculated as the number heterozygous genotypes divided by the number of nonreference homozygous genotypes in an individual

Among the 30 individuals for which genomes were sequenced, the average genome coverage ranged from 14 × to 41x, with a 25 × mean and 24 × median coverage (Fig. 1a). A total of 22,548,028 variants were identified, of which 18,876,115 were SNPs and 3,671,913 were indels. A vast majority of the genotypes were biallelic (92.2%) among which 98.2% of SNPs and 61% of indels were biallelic, wherein the reference allele is one of the two alleles. 1,154,779 SNPs and 304,467 indels were novel compared to the known variants in the *B. taurus* genome maintained by the 1000 Bull Genomes consortium. Among the 30 individuals, the mean was 6,500,780 SNPs per individual, ranging from 5,682,119 to 7,735,562 with a standard deviation (SD) of 335,872 SNPs.

The mean number of indels was 782,237 ranging from 686,334 to 924,185 with a SD of 39,517 indels. Furthermore, the number of sites for which no variants were called (i.e., missing variants) ranged from 100,127 to 217,928 (Fig. 1b) with a SD of 28,153. Overall, a variant was identified every 0.1 kb of the genome. Finally, the number of nonreference homozygous variants per individual ranged from 2,003,231 to 4,065,446 (Fig. 1d) with an average of 3,031,476 and SD of 331,011 while the number of heterozygous variants ranged from 2,879,108 to 7,331,882 (Fig. 1c) with a mean of 4,868,911 and a SD of 716,995. Accordingly, the heterozygosity ratio (HR) calculated as the number of heterozygous genotypes divided by the number of nonreference homozygous genotypes was on average 1.7. Although HR ranged from a minimum of 0.7 to maximum of 3.7 (Fig. 1e), the mode for the 30 individuals was 1.6 with a SD of 0.5.

### Summary of predicted biological consequences of variants

To better characterize the potential consequences of the identified mutations, we annotated the variants with snpEff and predicted the effects of variation on genes (Table 2). A large majority of the variants (88.85%) were in the intergenic and intronic regions, 42.56% and 46.29% respectively. Less than 1% (313,756) were in the exonic regions and much less (0.03% or 10,527) were in the splice donor/acceptor sites (Table S1). For the protein coding regions 152,892, 128,821 and 1,715 SNPs were identified as silent, missense and nonsense mutations. Overall, about 35 million putative effects were predicted for the roughly 22 million variants and were annotated into sequence ontology classes using the snpEff (Table 2, Table S1). The 35 million predicted effects were classified into impact categories as follows; (a) High impact (19,096): The variant is assumed to be deleterious to protein function, such as missense or nonsense SNPs, (b) Moderate impact (132,053): A nondisruptive variant that may change protein effectiveness, (c) Low impact (178,466): Assumed to be harmless, such as silent mutations, (d) Modifier (32,319,811): There is no evidence of impact, or it is difficult to predict effects of non-coding variants with the data at hand.

**Table 2:** Distribution of types of effects of the variants predicted by snpEff, a complete list is found in Table S1

Type	Count	Percent
Disruptive inframe deletion	1,340	0.00%
Disruptive inframe insertion	823	0.00%
Exon loss variant	8	0.00%
Frameshift variant	6,321	0.02%
Missense variant	128,377	0.36%
Splice acceptor variant	6,472	0.02%
Splice donor variant	4,356	0.01%
Splice region variant	34,945	0.10%
Intron variant	16,382,594	46.29%
other	18,828,998	53.20%
Total	35,394,234	100%

### Population stratification and genomic diversity

**Fig. 2:**
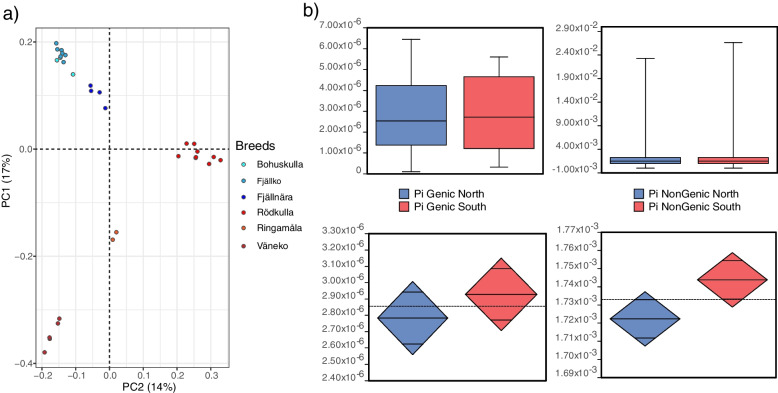
Population stratification and genetic diversity. Legend: **a** Principal component analysis (PCA) projection based on 19 million biallelic autosomal SNPs. The first two principal components (PC1 and PC2) together explain 31% of the total variance. **b** Nucleotide diversity (π) of the genic and non-genic regions of autosomes

To determine the structure of the population, we performed a PCA of ~ 19 million genome wide biallelic SNPs and plotted the genotype covariation along the first two principal components PC1 and PC2 (Fig. 2a). PC1 (y axis), which explained ~ 17% of the total variation, separated the northern breeds (Fjäll, Fjällnära and Bohuskulla) from the southern breeds (Rödkulla, Ringamåla and Väneko). PC2, which explained an additional 14% of the total variation, separated the subpopulations within the two groups. In addition to the whole genome cohort of ~ 19 million SNPs, we also performed PCA on four randomly sampled subsets of 100,000 SNPs (Additional file 1). The population stratification in all the random subsamples were consistent with that of the complete cohort of 19 million SNPs. Additionally, these results are consistent with earlier studies based on SNP array data, with an average SNP spacing of 19 kb [4, 8].

In general, the genetic differentiation among the northern breeds was lower compared to the southern breeds. The southern breeds, Rödkulla, Ringamåla and Väneko were well separated whereas the separation of the northern breeds was less clear. Although the Fjällko and Fjällnära subpopulations were separated, the genetic differentiation between Fjällko and Bohuskulla was low.

To better understand the extent of genetic differentiation between the northern and southern populations, we quantified pairwise nucleotide diversity (π) in the genic and nongenic regions of autosomes (Fig. 2b). Overall π values were three to four orders of magnitude higher in teh non-genic regions (ranging from about 1 × 10^–3^ to 2.5 ξ 10^-2^) compared to the π values in genic regions (ranging from 0.1 × 10^–6^ to 6.5 × 10^–6^). Consistent with the higher genetic differentiation among the southern breeds, the average π was higher in both genic and non-genic regions. However, significant difference in the mean π was observed only in the nongenic regions (t-test, *P* < 0.05), which is the bulk of the genome.

### Nuclear DNA genealogy and recent maternal ancestry

**Fig. 3:**
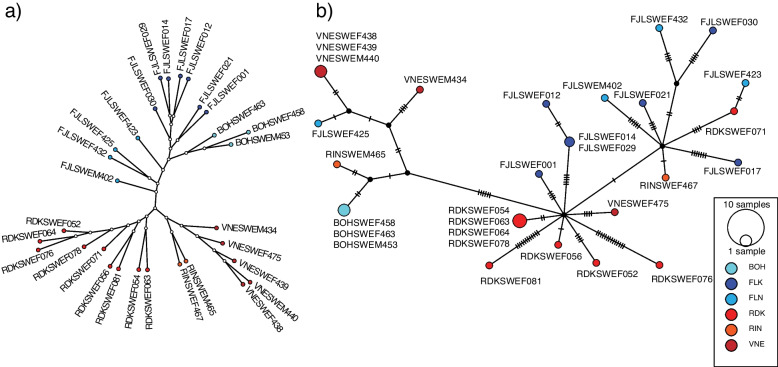
Nuclear DNA genealogy and maternal ancestry. Legend: **a** Whole genome NJ tree estimated from ~ 19 million autosomal SNPs. **b** Median joining haplotype network based on complete mitochondrial genome. Most nodes represent one individual, size of larger nodes corresponds to multiple individuals with identical haplotypes. The number of mutations is drawn as hatch marks along edges. In both (**a**) and (**b**) nodes that correspond to the northern breeds are colored in shades of blue and nodes corresponding to southern breeds are colored in shades of red. Internal nodes (black) are inferred ancestral sequences

A more detailed pattern of the genetic divergence amongst the breeds is shown in the genealogy (Fig. 3a). A NJ tree was estimated from the genetic distances calculated from ~ 19 million autosomal SNPs. Identical trees were produced from observed distance (uncorrected P distance) and from distances estimated from two different nucleotide substitution modes, namely GTR and TN distances as well as a coalescent model using SVD quartets analysis. The relationships within and between the breeds were mostly consistent with the relatedness observed in the PCA projection, such that the northern and southern clusters are distinct. The two clusters also distinguished the southern breeds with mainly red coat color from the mostly white colored northern breeds. The red breeds shared a common ancestor, and among the red breeds, Ringamåla were more closely related to Väneko, than they were to Rödkulla. Within the northern breed group, Bohuskulla was more closely related to Fjällko than to Fjällnära. However, in the sequenced samples, individuals of Fjällnära breed did not share a common ancestor, even though they are known to be closely related to Fjällko. The sequenced samples of Fjällnära were from different founding herds of the breed and our results show that the founding herds were genetically differentiated.

Since 25 of the 30 of sequenced individuals were cows and maternal ancestry is rarely assessed using WGS methods, we constructed a haplotype network based on complete mitochondrial (mt) genomes to trace maternal lineages of the sequenced individuals. The 16.34 kb mtDNA was sequenced at a very high average coverage of 500x. The nucleotide diversity was much lower (π = 1.6 × 10^–7^) compared to nuclear DNA. We identified a total of 94 segregating sites out of 130 variants identified. In the median joining network in Fig. 3b, the maternal lines of descent inferred from mtDNA were inconsistent with the nuclear DNA based genealogy (Fig. 3a), and the recorded demographic origins of the breeds. For instance, mtDNA was identical in all three samples of Bohuskulla, but was most closely related to one of the Ringamåla hapolotypes (RINSWEM465) and was more closely related to 4 out of 5 Väneko haplotypes than to Fjällko and Fjällnära as seen in the relatedness based on nuclear DNA (Fig. 3a).

A closer examination of pedigrees in the breed database (https://db.Fjallko.se) showed that the two Fjällko individuals (FJLSWEF014 and FJLSWEF029) that had identical mtDNA haplotype were closely related, FJLSWEF014 was the maternal grandmother of FJLSWEF029. All the other Fjällko samples had distinct mtDNA haplotypes. The mtDNA of one individual, FJLSWEF001 was similar to several Rödkulla, but her maternal ancestors can be traced back about 90 years and were all recorded as Fjällko. Her oldest maternal ancestor was a cow called Lärka with unknown birth year and place but that had a daughter born 1934 that lived in Burträsk in the north-east of Sweden.

Four Rödkulla had identical mtDNA haplotypes. From the pedigree data we could see that they all descended on the maternal side from Norwegian Eastern Red Polled cows imported from a herd in Sarpsborg, located in south eastern Norway quite close to the Swedish border. One of the Rödkulla RDKSWEF071 had a maternal ancestor four generations back that was imported from Norway. The mtDNA of RDKSWEF071 only differed by two mutations from the mtDNA of the Fjällnära from Funäsdalen (FJLSWEF423). Since Funäsdalen is close to the Norwegian border, it is possible that the farm at Funäsdalen also bought cows from Norway at some point in the past.

Three of the five Väneko had identical mtDNA, and these three: VNESWEF438, VNESWEF439, VNESWEF440, represented three different cow lines according to information provided by the breed organisation. These cow lines thus had a common maternal ancestor. The other two mtDNA haplotypes in Väneko represented two other cow lines. One of the Fjällnära mtDNA haplotypes was similar to the mtDNA that was present in three Väneko samples (3 mutations differed). It was found in a Fjällnära cow with origin from the founder herd Klövsjö. The other three Fjällnära mtDNA haplotypes were spread out in the network (Fig. 3b), i.e., related to different ancestors of other breeds.

Finally, the two Ringamåla haplotypes found in the data were very different from each other. They were from two different cow lines and these two cow lines were thus not closely related on the maternal side.

### Verification of predicted consequences

It is not clear if the variants and snpEff predicted variant-impacts reported in the summary (Table 2) are: (1) widespread in the population for which variants are identified or if it is present in only one individual in the population, and (2) if predicted impacts produce/affect a phenotype. To verify the above uncertainties we further analyzed genes responsible for coat color phenotypes since color traits are known to have been targets of selection in several of the studied breeds and there are differences between our studied breeds in coat color phenotypes. Polymorphism in the genes involved in the melanin biosynthesis pathway are known to be associated with variation in coat color. To further investigate variants in coat color genes, we first identified the variant effect predictions and the potential impact of the identified variants in our samples. A summary of the variant effects and predicted impact for coat color genes in our samples is shown in Table 3. Many genes that are known to be associated with coat color variation are involved in the melanin biosynthesis pathway to produce the coloring pigments eumelanin and pheomelanin. These are *ASIP, DCT, KIT, MC1R, PMEL, TYR and TYRP1*. High impact mutations, identified here, were predicted in *KIT* and *MC1R,* both of which are hormone receptors, which stimulate downstream processes of the melanogenesis pathway. Loss or modification of function of these proteins are known to affect pigment production.We then determined the prevalence of the *MC1R* and *KIT* high impact mutations in our samples. For this, the DNA sequence for each gene for each individual was extracted and variants were further analyzed to characterize the high impact mutations.

**Table 3:** Summary of variant-impact and type of variant in genes associated with coat color phenotype

Variant	ASIP	DCT	KIT	MC1R	PMEL1	PMEL2	TYR	TYRP1
*Impact*								
HIGH impact	0	0	1	2	0	0	0	0
MODERATE impact	0	6	3	1	4	4	2	3
LOW impact	1	10	7	0	1	0	3	4
MODIFIER	18	538	550	2	22	23	775	59
*Type*								
3_prime_UTR_variant	1	6	10	2	1	0	2	4
5_prime_UTR_variant	0	4	1	0	0	0	0	0
disruptive_inframe_deletion	0	0	0	0	1	1	0	0
downstream_gene_variant	0	0	0	0	0	1	0	0
frameshift_variant	0	0	0	**2**	0	0	0	0
intron_variant	17	530	541	0	21	18	773	55
missense_variant	0	6	3	1	3	3	2	3
splice_acceptor_variant	0	0	**1**	0	0	0	0	0
splice_region_variant	0	3	2	0	0	0	0	0
synonymous_variant	1	8	6	0	1	0	3	4
upstream_gene_variant	0	0	0	0	0	4	0	0

PMEL1 and PMEL2 are distinct transcripts of the PMEL gene

### MC1R

*MC1R* codes for the melanocortin receptor type 1, a relatively small, intron-less gene with a single 951 bp exon located on chromosome BTA18. MC1R encodes for 317 aa protein of the G protein-coupled receptor family. We identified three alleles in our samples and the first two of them are listed in OMIA [26]: (1) The missense mutation is a single C—> T substitution at pos. 296 causing a L99P mutation. This allele was present in 4 out of 14 individuals of the northern breeds but not in the southern breeds. The *C* allele at this position is known as the dominant black allele *E*^*D*^ [27, 28]. (2) The first frameshift variant caused by a G nucleotide deletion at pos. 310/311, results in a premature stop codon in place of tyrosine at pos. 155 in the protein. This allele was present in 9 out of 14 individuals in the northern breeds and 13 of in total 16 samples in the southern breeds. This deletion is known as the recessive red allele *e* [27]. (3) A second frameshift variant caused by a T nucleotide deletion at pos. 692 was found in only 2 samples of the southern breed Rödkulla (RDKSWEF063 and RDKSWEF076).

### KIT

*KIT* is a Tyrosine kinase receptor, also known as the proto-oncogene c-KIT. The bovine *KIT* homolog is 87.35 kb with 21 exons that code for a 977 amino acid protein [29]. We identified a variant at position 72,813, that is likely to disrupt splicing of exon 8/9. In addition, we found a 3-bp deletion in the *KIT* gene in 4 of our samples: Two Fjällko (FJLSWEF001, FJLSWEF029) and two Väneko (VNESWEF438, VNESWEM440) samples. It is not known if this deletion has any impact on KIT activity or if it is associated with a phenotype.

## Discussion

Highly endangered livestock populations are at risk of suffering from inbreeding and/or genetic drift all over the world. Swedish native cattle breeds are known to have adapted to their local habitat characterized for example by cold winters, and seem to have been selected for disease resistance among other characteristic traits of importance for breeding and conservation programs [3]. This is the first WGS-based genetic analysis of indigenous Swedish cattle. We sequenced genomes of 30 individuals representing six Swedish native cattle breeds, with medium to high coverage (14x—45x) to assess the genetic diversity of these native breeds in more detail compared with previous SNP-based studies [3, 4, 8]. The average nucleotide diversity (π = 1.6 × 10^–3^) is on par with that reported for other native European breeds [20, 30, 31]. However, we were able to identify a higher number, about 22.5 million variants in 30 samples, compared to about 14–7.5 million variants identified in about 10–50 samples in other European breeds [20, 30, 31]. The high number of identified variants despite the small population sizes of the native Swedish breeds is an important result that shows that these breeds harbour a lot of genetic diversity that could be very importnat for future cattle breeding.

Accordingly, we identified 1,154,779 novel SNPs and 304,467 novel indels compared to other currently known variants identified in various breeds across the globe by the 1000 Bull Genomes consortium. WGS of larger populations is likely to reveal more novel variants. However, a more detailed characterization of the variants, including transcriptomic and proteomics analyses, will be necessary to evaluate if these novel variants are deleterious or beneficial. Identifying deleterious variants will be useful for designing SNP-arrays for routine genetic screening of individuals to identify disease risk factors, for selective breeding and preservation programs.

Earlier studies have shown that it is possible to characterize the genetic diversity of larger populations using SNPchip technology [32]. A weakness on our present study was the low number of animals per breed. However, the overall pattern of diversity and relatedness between the breeds in the present study was similar as in the previous SNP-based studies of these six Swedish native cattle breeds [3, 4]. For example, similar results can be seen when comparing the PCA results Fig. 2a in the current study with those in Fig. 3a in the study by Upadhyay et al. [4]. The lower cost of using SNP genotyping allowed for a larger sample size per breed in the study by Upadhyay et al. [4], and thereby better estimates of population parameters such as LD and distribution of ROH than would be possible with WGS data. However, many variants associated with desirable phenotypes were not covered in the SNP arrays due to the relatively low coverage of the genome: SNP discovery rate of 1 SNP/20 kb using SNPchip-based genotyping compared to 1 SNP/0.2 kb using WGS-based genotyping. For instance, previously characterized polymorphisms in genes associated with coat color variation were not included in the commercially available SNP arrays used for routine genotyping. In contrast, we were able to identify and characterize the prevalence of these coat color polymorphisms in our samples. Our sequence data can also be used to identify additional variants associated with other phenotypes in these breeds in the future.

We were able to verify computationally predicted consequences of missense and frameshift mutations in some genes known to associated with Mendelian traits. In particular, we identified polymorphisms in key genes that are associated with the variation in coat color phenotypes. MC1R plays a crucial role in the melanogenesis pathway for pigmentation in hair and skin color in humans and coat color in animals. *MC1R* variants are associated with black or red coat color variation, which depends on two polymorphic sites [28]. These two polymorphisms correspond to three alleles *E*^*D*^, *E*^+^ and *e*. Missense mutation derived E^D^ (p.99Pro) and E^+^ wild type (p.99Leu) alleles are responsible for black coat color and a combination of red or reddish brown/black coat colors, respectively [26, 28]. A frameshift mutation caused by deletion of guanine nucleotide at position 310/311, which introduces a premature stop codon and is known as the recessive red mutation *e* [27] is seen more frequently in the southern (Red) breeds in our samples. Since this mutation results in the production of pheomelanin, but not eumelanin, it is associated with red coat colors, while the former is associated with black coat colors. We found that red coat color, regardless of speckled or sided phenotypes and degree of spotting, is associated with a premature stop codon in *MC1R* caused by a G nucleotide deletion at pos. 310/311 known as the recessive red mutation *e*. This frameshift mutation overrides other relatively less deleterious frameshift and missense mutations in *MC1R*. Furthermore, this or similar loss-of-function variants, which biases the production of pheomelanin over eumelanin may be a major cause of red coat color in general. Further, large-scale analyses of Red breeds is necessary to verify our prediction.

In contrast to the *MC1R* SNVs, *KIT* variants that are associated with the dominant white coat color and color-sided phenotypes as well as gonadal hypoplasia are structural variants [33–35]. All the mentioned phenotypes are observed in the northern breeds Fjällko and Fjällnära. The best characterized structural variant is the duplication and translocation of *KIT* to BTA29 from BTA6 [29]. In this study we did not analyze structural variants, but the HIGH impact variant identified here are likely to be deleterious to the expression of a functional KIT protein. Malfunctioning KIT is known to result in a lack of melanocytes, the melanin producing cells rather than altered pigment production effected by deletion of MC1R [28]. In the future, studies of structural variants and their association to phenotypes would be very interesting in these breeds.

Likewise, for a majority of the novel variants identified here, prior information is unlikely to be available. Therefore, most of the predictions should be treated with caution until further experimental verification. Besides, a more comprehensive WGS based survey would be necessary to better estimate the prevalence of the predicted high impact variants in indigenous populations. This can be accomplished by genomic as well as transcriptomic analysis of existing animals from native cattle breeds, rather than frozen samples. At any rate, the predictions could prove useful in designing better and/or custom SNP-arrays for large-scale genotyping studies and genetic screening for deleterious mutations.

The complex genealogy of the individuals that was detected in this study also show the added value of using WGS of animals compared with earlier SNP-array based studies [4, 8]. For example, it has long been known that Bohuskulla is closely related with the northern breeds in spite of its location in south-west Sweden [6, 36]. The breed is said to originate from local cattle from Kynnefjäll, but Fjällko or Fjällnära bulls have been used to some extent in breeding of Bohuskulla (http://allmogekon.se/bohuskulla/). The findings in this study support that the maternal ancestry is more similar to other native southern Swedish breeds like Väneko and Ringamåla. We also observed other examples where the maternal genealogy based on mtDNA did not match the relatedness based on whole-genome-data which indicates that the maternal and paternal ancestries are different.

## Conclusions

Our results show that a higher genetic diversity is prevalent in Swedish native cattle compared to commercial breeds despite the low population sizes. This is in agreement with several other studies of indigenous European breeds, which have adapted to harsh weather in the northern European regions. We predicted effects of the variants including SNPs and indels on protein coding regions and verified the potential effects on Mendelian traits like coat color. Because populations of many indigenous breeds are declining rapidly and that the variant discovery rate is significantly lower using the more common methods of large-scale genotyping, a more comprehensive WGS-based survey of genetic diversity is an urgent priority. Recording the genomic diversity and reconstructing the recent ancestry of the indigenous breeds are valuable to both the ongoing efforts of conservation of indigenous diversity and for imputation-based predictions of variant effects towards improving genomic selection programs.

### Supplementary Information

**Additional file 1: Figure S1.** Population stratification of Swedish native cattle breeds. Principal component analysis (PCA) projections based on four independent and random samplings of 100,000 biallelic autosomal SNPs.

**Additional file 2: Table S1.** Summary statistics of the types of effects of the variants predicted by snpEff. Approximately 35 million effects were predicted for the 22 million genotypic variants and annotated into sequence ontology classes.

## Data Availability

Sequence data for these samples are included in the 1000 Bull Genomes Project and we have also made the whole-genome sequence data available in the European Nucleotide Archive with project accession PRJEB60564.
