# Supplementary material for: Genetic diversity and recent ancestry based on whole-genome sequencing of endangered Swedish cattle breeds
Source: BMC Genomics. 2024 Jan 22;25:89. doi: 10.1186/s12864-024-09959-9 (PMC10802049; doi:10.1186/s12864-024-09959-9)
Supplement: Supplementary file 1 — Additional file 1: Figure S1. Population stratification of Swedish native cattle breeds. Principal component analysis (PCA) projections based on four independent and random samplings of 100,000 biallelic autosomal SNPs. [file 12864_2024_9959_MOESM1_ESM.pdf]

Additional File 1: Figure S1

Population stratification of Swedish native cattle breeds

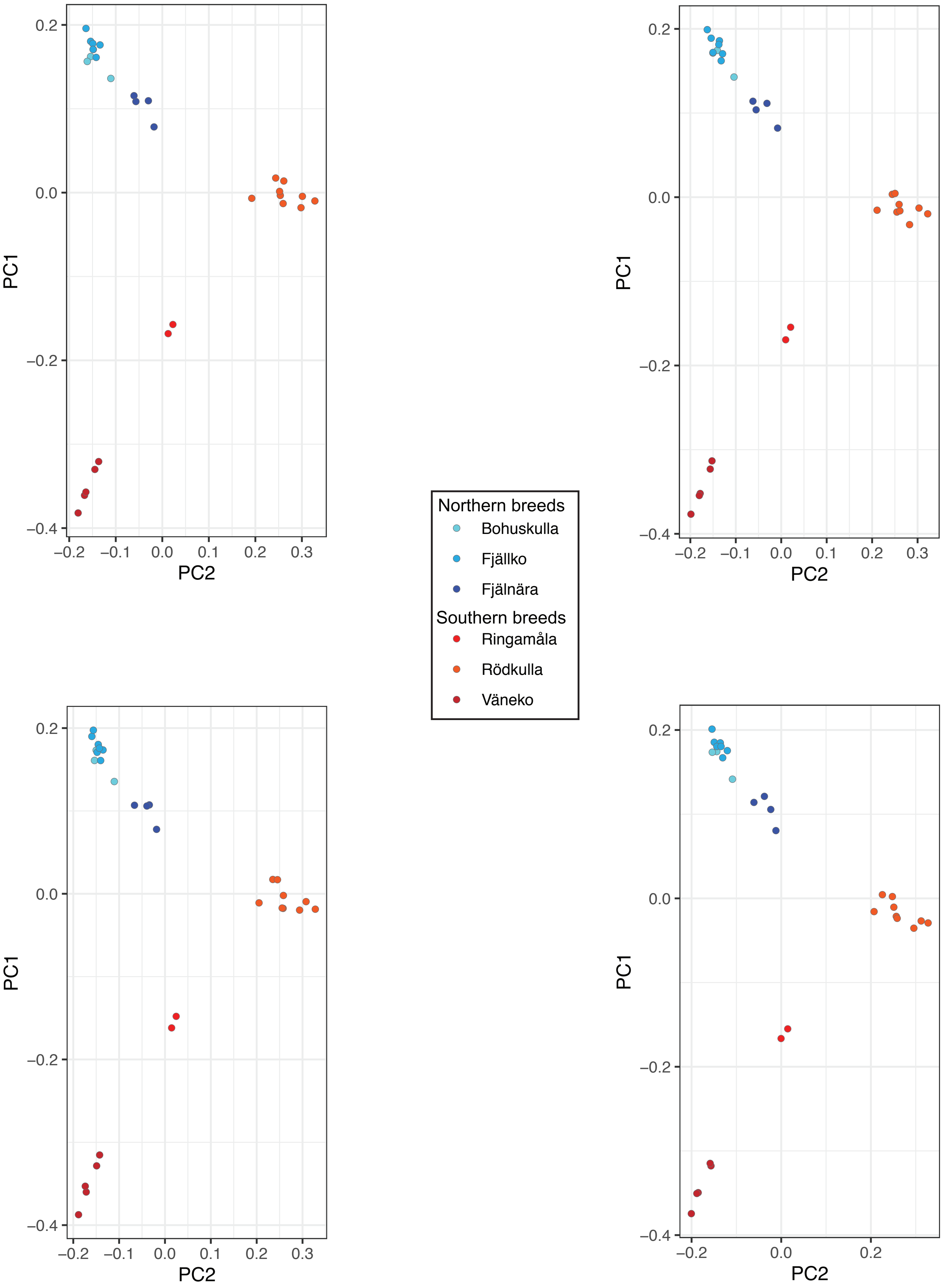

Title: Population stratification of Swedish native cattle breeds

Description: Principal component analysis (PCA) projections based on four independent and random samplings of 100,000 biallelic autosomal SNPs.
