# Supplementary material for: Genetic diversity and recent ancestry based on whole-genome sequencing of endangered Swedish cattle breeds
Source: BMC Genomics. 2024 Jan 22;25:89. doi: 10.1186/s12864-024-09959-9 (PMC10802049; doi:10.1186/s12864-024-09959-9)
Supplement: Supplementary file 2 — Additional file 2: Table S1. Summary statistics of the types of effects of the variants predicted by snpEff. Approximately 35 million effects were predicted for the 22 million genotypic variants and annotated into sequence ontology classes. [file 12864_2024_9959_MOESM2_ESM.pdf]

**Table S1. Summary statistics of the types of effects of the variants predicted by snpEff**

| Type | Count | Percent |
|---|---|---|
| 3_prime_UTR_truncation | 1 | 0% |
| 3_prime_UTR_variant | 117,583 | 0.33% |
| 5_prime_UTR_premature_start_codon_gain_variant | 5,686 | 0.02% |
| 5_prime_UTR_truncation | 1 | 0% |
| 5_prime_UTR_variant | 45,521 | 0.13% |
| bidirectional_gene_fusion | 17 | 0% |
| conservative_inframe_deletion | 769 | 0.00% |
| conservative_inframe_insertion | 836 | 0.00% |
| disruptive_inframe_deletion | 1,340 | 0.00% |
| disruptive_inframe_insertion | 823 | 0.00% |
| downstream_gene_variant | 1,708,598 | 4.83% |
| exon_loss_variant | 8 | 0% |
| frameshift_variant | 6,321 | 0.02% |
| gene_fusion | 26 | 0% |
| initiator_codon_variant | 19 | 0% |
| intergenic_region | 15,063,491 | 42.56% |
| intragenic_variant | 2 | 0% |
| intron_variant | 16,382,594 | 46.29% |
| missense_variant | 128,377 | 0.36% |
| non_coding_transcript_exon_variant | 23,909 | 0.07% |
| non_coding_transcript_variant | 132 | 0% |
| splice_acceptor_variant | 6,472 | 0.02% |
| splice_donor_variant | 4,356 | 0.01% |
| splice_region_variant | 34,945 | 0.10% |
| start_lost | 267 | 0.00% |
| start_retained_variant | 8 | 0% |

| | | |
|---|---|---|
| **stop_gained** | 1,873 | 0.01% |
| **stop_lost** | 270 | 0.00% |
| **stop_retained_variant** | 132 | 0% |
| **synonymous_variant** | 152,781 | 0.43% |
| **transcript_ablation** | 10 | 0% |
| **upstream_gene_variant** | 1,707,066 | 4.82% |
| **Total** | 35,394,234 | 100% |

Approximately 35 million effects were predicted for the 22 million genotypic variants and annotated into sequence ontology classes.
